# Genetic and toxinological divergence among populations of *Tityus trivittatus* Kraepelin, 1898 (Scorpiones: Buthidae) inhabiting Paraguay and Argentina

**DOI:** 10.1371/journal.pntd.0008899

**Published:** 2020-12-14

**Authors:** Adolfo Borges, Antonieta Rojas de Arias, Sabrina de Almeida Lima, Bruno Lomonte, Cecilia Díaz, Carlos Chávez-Olórtegui, Matthew R. Graham, Evanguedes Kalapothakis, Cathia Coronel, Adolfo R. de Roodt

**Affiliations:** 1 Centro para el Desarrollo de la Investigación Científica (CEDIC), Asunción, Paraguay; 2 Laboratorio de Biología Molecular de Toxinas y Receptores, Instituto de Medicina Experimental, Facultad de Medicina, Universidad Central de Venezuela, Caracas, Venezuela; 3 Laboratorio de Inmunoquimica, Departamento de Bioquímica e Inmunologia, Instituto de Ciências Biológicas, Universidade Federal de Minas Gerais, Belo Horizonte, Brasil; 4 Instituto Clodomiro Picado, Universidad de Costa Rica, San José, Costa Rica; 5 Department of Biology, Eastern Connecticut State University, Willimantic, Connecticut, United States of America; 6 Departamento de Biologia Geral, Instituto de Ciências Biológicas, Universidade Federal de Minas Gerais, Belo Horizonte, Brasil; 7 Instituto Nacional de Producción de Biológicos “Carlos G. Malbrán”, Buenos Aires, Argentina; Instituto Butantan, BRAZIL

## Abstract

Envenoming by scorpions in genus *Tityus* is a public health problem in Tropical America. One of the most medically significant species is *Tityus trivittatus*, which is known to occur from southwest Brazil to central-northern and eastern Argentina. In this work, we studied the lethality, composition, antigenicity, and enzymatic activity of venom from a *T*. *trivittatus* population found further north in urban areas of eastern Paraguay, where it has caused serious envenomation of children. Our results indicate that the population is of medical importance as it produces a potently toxic venom with an LD_50_ around 1.19 mg/kg. Venom neutralization in preliminary mouse bioassays was complete when using Brazilian anti-*T*. *serrulatus* antivenom but only partial when using Argentinean anti-*T*. *trivittatus* antivenom. Venom competitive solid-phase enzyme immunoassays and immunoblotting from Argentinean and Paraguayan *T*. *trivittatus* populations indicated that antigenic differences exist across the species range. SDS-PAGE showed variations in type and relative amounts of venom proteins between *T*. *trivitattus* samples from Argentina and Paraguay. MALDI-TOF mass spectrometry indicated that while some sodium channel toxins are shared, including β-toxin Tt1g, others are population-specific. Proteolytic activity by zymography and peptide identification through nESI-MS/MS also point out that population-specific proteases may exist in *T*. *trivitattus*, which are postulated to be involved in the envenoming process. A time-calibrated molecular phylogeny of mitochondrial COI sequences revealed a significant (8.14%) genetic differentiation between the Argentinean and Paraguayan populations, which appeared to have diverged between the mid Miocene and early Pliocene. Altogether, toxinological and genetic evidence indicate that *T*. *trivitattus* populations from Paraguay and Argentina correspond to distinct, unique cryptic species, and suggest that further venom and taxonomic diversity exists in synanthropic southern South American *Tityus* than previously thought.

## Author summary

Scorpionism (the medical consequence of scorpion stings in humans) is a neglected health problem in tropical and subtropical areas associated with poverty. This study is the first to compare venoms among core (Argentinean) and peripheral (Paraguayan) populations of the noxious *Tityus trivittatus*, the most medically important scorpion in the southernmost section of South America. The work demonstrated the lethality of the venom of urban populations of *T*. *trivittatus* in Paraguay, where it has caused severe cases in children. We obtained data indicating that there are significant differences in venom composition and function, and also recognition by therapeutic antivenoms available in the region, among these Argentinean and Paraguayan scorpion populations which historically have been assigned to the same species. Our genetic study revealed that in fact these two populations diverged between ~15–5 Million years ago, indicating they are distinct species. These results indicate that southern South American scorpions in the genus *Tityus* which co-distribute with humans are more diverse in terms of their venoms and species composition than previously thought, and that further studies are warranted to design more effective therapeutic tools against scorpionism in the region to tackle such diversity.

## Introduction

Envenoming by scorpions belonging to the genus *Tityus* is a public health problem in southern South America, which has been classified as a hyperendemic area of scorpionism [1,2]. In southeast Brazil the most problematic scorpion is *T*. *serrulatus* Lutz & Mello, a parthenogenetic species currently expanding its range and responsible for most severe envenomations in the area [3]. The second most medically important scorpion in the region is *T*. *trivittatus* Kraepelin, a species responsible for the majority of severe scorpion envenomations in Argentina, mostly in children [4,5]. The species’ range extends from central-northern and eastern Argentina to eastern Paraguay and southeast Brazil, and has been predicted to increase in response to ongoing global climate change [6].

Venoms from *T*. *serrulatus* and *T*. *trivittatus* contain low molecular mass toxins that affect the gating mechanism of various voltage-sensitive ion channels [7]. The main lethal toxins in *Tityus* venoms are sodium channel (Nav)-active toxins (NaTxs), which affect either the activation or inactivation components of sodium channel currents in excitable cells, producing sustained depolarization and massive discharge of neurotransmitters [8]. Rapid tissue distribution of these toxins has resulted in high mortality rates in children under 10 years of age, so severe stings require prompt treatment with specific antivenoms and intensive cardio-respiratory support [9].

The medical significance of *T*. *trivittatus* was unknown outside Argentina until recently, when severely envenomed children were reported from eastern Paraguay. These cases presented with psychomotor agitation, profuse sweating, serum hypokalemia, and altered cardiac frequency as a consequence of left ventricular dysfunction [10]. Unlike Argentinean populations of *T*. *trivittatus*, which are parthenogenetic [11], those that are common in urban areas of eastern Paraguay, including the capital city of Asunción exhibit sexual dimorphism [10,11]. Venom from Argentinean populations has been thoroughly studied from clinical, immunological, biochemical, pathological and toxicological perspectives [12,7,4]. However, considering the potential medical importance of *T*. *trivittatus* outside Argentina, its predicted changing distribution due to global warming, and the reported divergence in venom composition and action even among closely related *Tityus* species [1], further study is urgently needed. In this contribution, we studied the lethality, neutralization by available antivenoms, proteolytic activity, and molecular mass fingerprinting of venom from an urban *T*. *trivittatus* population from Paraguay. Our study revealed significant toxinological and genetic divergence between the Paraguayan samples and *T*. *trivittatus* from Argentina, indicating that they probably comprise unique cryptic species.

## Methods

### Ethical statement

The Animal Research Ethics Committee of the Centro para el Desarrollo de la Investigación Científica reviewed the study protocol involving mice for toxicity and neutralization assays on 02/04/2019 and approved the research (approval code: 01/2019). The institutional Animal Research Ethics Committee follows the guidelines for animal research established by the United States National Research Council (https://grants.nih.gov/grants/olaw/guide-for-the-care-and-use-of-laboratory-animals.pdf).

### Scorpion venoms

**Fig 1:**
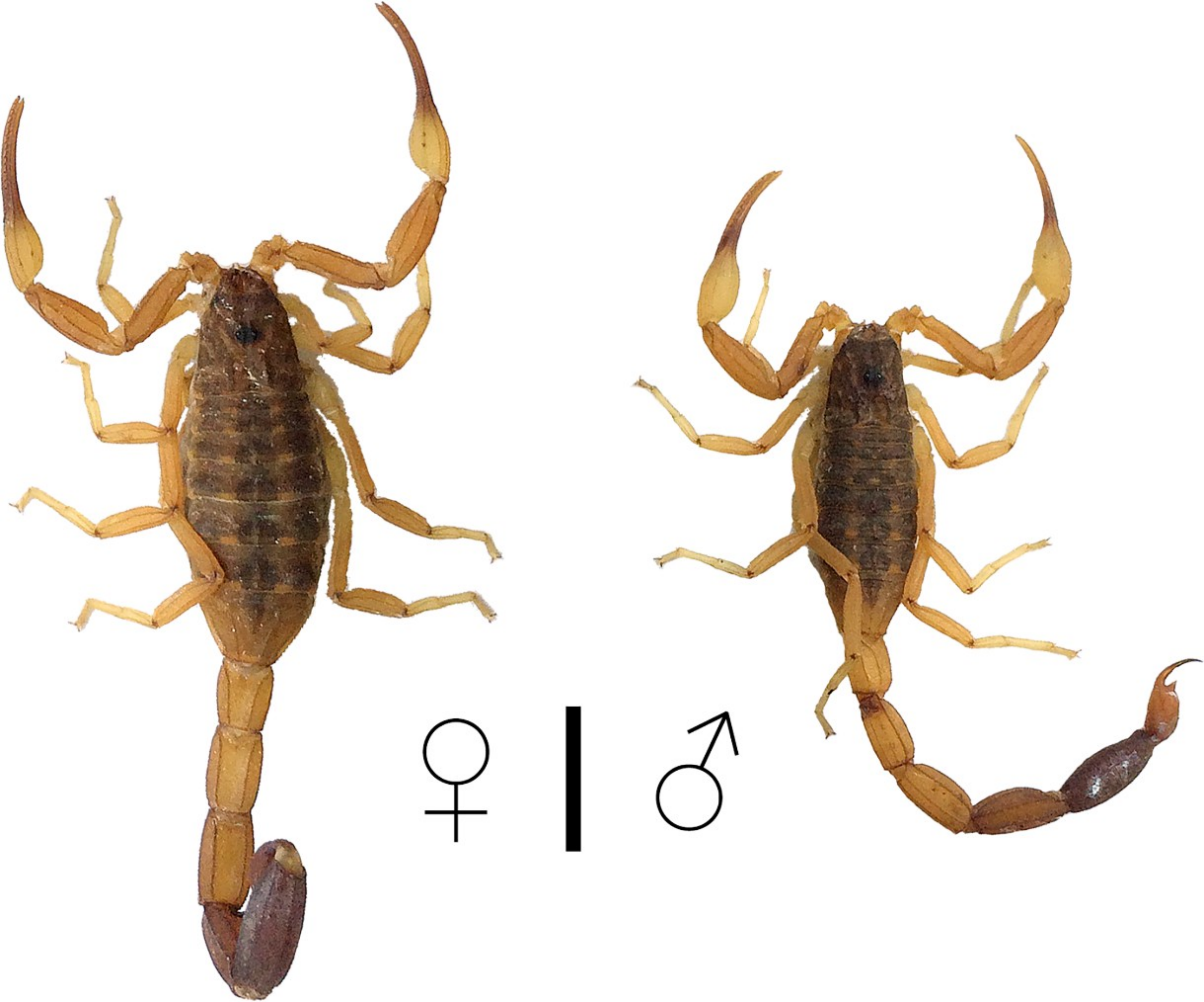
Habitus of female and male specimens of *Tityus trivittatus* collected in urban areas of Asunción, Paraguay. Bar, 1 cm. Photographs by A. Borges.

Scorpions from Paraguayan population of *T*. *trivittatus* (Fig 1) were collected from crevices and pipelines at human dwellings within the urban area of Asunción, Paraguay. Live specimens were transferred to the lab where they were housed with water *ad libitum* and fed with crickets (*Acheta domesticus*). Venom was extracted from male and female scorpions by electrical stimulation of the telson following procedures in [13] and lyophilized at -50°C and 80 mBar of pressure. Prior to *in vivo* or *in vitro* studies, lyophilized samples, containing an equal proportion of venom from male and female specimens, were dissolved in either phosphate-buffered saline (PBS, 137 mM NaCl, 2.7 mM KCl, 8 mM Na_2_HPO_4_, and 2 mM KH_2_PO_4_) or doubly distilled water, respectively. Venom from the Argentinean population of *T*. *trivittatus* was from specimens collected in Paraná, Entre Ríos. Venom from *T*. *serrulatus* was also obtained by electrical stimulation of the telson of specimens collected in Belo Horizonte, Minas Gerais, Brasil. Venom from *T*. *discrepans* was obtained electrically from specimens collected in San Antonio de los Altos, Miranda, Venezuela. Venom protein content was estimated by the Lowry method [14].

### Venom Lethality (LD_50_)

The toxicity of crude venom from *T*. *trivittatus* (Paraguay) was assessed using NIH Swiss mice weighing 20–22 g obtained from the Instituto de Investigaciones en Ciencias de la Salud, Asunción, Paraguay. Mice (four animals per dose) were injected intraperitoneally (i.p.) with the following doses (in venom mg/kg body weight): 2.48, 1.66, 1.10, 0.74, and 0.49. A control group received the same injection (0.2 mL) of PBS. Mice were observed for 48 h after injection for symptoms of intoxication and death. Median lethal dose (LD_50_) values (in mg/kg) and corresponding 95% confidence intervals (CI) were calculated using Probit analysis according to the Spearman-Karber method [15]. Animal manipulations were performed according to the regulations of the Centro para el Desarrollo de la Investigación Científica (CEDIC), Asunción, Paraguay (section 2.13).

### *In vivo* neutralization tests

A preliminary *in vivo* assessment of the neutralization capacity of scorpion therapeutic antivenoms (AVs) was conducted using a single dose of AV sufficient to neutralize 3–5 LD_50_ of the corresponding control venoms as indicated by the manufacturers [16,17]. Venom samples of *T*. *trivittatus* (Paraguay) containing 3×LD_50_ (LD_50_ = 23.8 μg/20 g mouse) were incubated for 1 h at 37°C with 100 μl of AVs (see below) mixed with 100 μl of PBS as suggested in standardized procedures [18]. After incubation, samples were injected (i.p.) into NIH Swiss mice (four animals per antivenom group) for the neutralization assay. Three control groups (four mice each) were used: a first group received a mixture of snake antivenom (Anti-(*Bothrops jararaca* + *Crotalus durissus terrificus*), Fundação Ezequiel Dias, Belo Horizonte, Brazil) and 3×LD_50_ of *T*. *trivittatus* (Paraguay) venom per mouse and incubated as above. A second group received PBS (200 μl) and 3×LD_50_ venom per mouse, incubated and injected as described above. A third group only received PBS. Two scorpion AVs were assayed: anti-*T*. *serrulatus* (Instituto Vital Brazil, Nitéroi, Brasil) (Batch 186001, expiration date 10/31/2021), and anti-*T*. *trivittatus* (Instituto Nacional de Productos Biológicos “Carlos G. Malbrán”, Buenos Aires, Argentina) (Batch L930, expiration date 07/31/2020). The procedure was repeated twice. Surviving mice were counted at 48 h. Protein concentration in AVs was measured by the Biuret method [19], using a Proti 2 protein determination kit (Wiener, Rosario, Argentina). All experiments were conducted before the expiration dates of the antivenoms.

### Cross-recognition by western blot

Electrophoresis of venoms in sodium dodecyl sulfate-polyacrylamide (SDS-PAGE) gels was carried out with 20% gels using Tris-Glycine as running buffer and stained with either Coomassie Brilliant Blue R-250 or silver staining, as outlined in [20]. For immunoblotting, venom samples (typically 10–15 μg protein) were solubilized in reducing sample buffer (BioRad), separated by SDS-PAGE, and subsequently transferred to nitrocellulose paper. Membranes were blocked with blocking buffer [1% (w/v) skimmed non-fat milk, 0.3% (v/v) Tween 20/PBS] for 1 h and then incubated with therapeutic sera from immunized horses, diluted in the same blocking buffer (1/1,000), for 1.5 h. Membranes were washed with 0.05% (v/v) Tween 20/PBS and incubated with goat anti-horse horseradish peroxidase-conjugated secondary antibody (Sigma) (diluted 1/50,000) in blocking buffer for 1 h. Membranes were washed once again and blots were developed using Luminata Forte Western HRP Substrate (Millipore).

### Solid-phase enzyme immunoassay (ELISA)

One hundred nanograms of venom protein each from Argentinean and Paraguayan *T*. *trivittatus* was adsorbed to the surface of separate sets of wells of MaxiSorp flat bottom microtitration plates (Nunc) at 4°C for 12–14 h, and blocked with 3% (w/v) bovine serum albumin containing 0.05% (v/v) Tween-20 for 3 h at room temperature. Plates were washed with saline containing 0.05% Tween-20 and then 100 μl of different AV (anti-*T*. *trivittatus*, Argentina) dilutions (1:200 to 1:51,200) were added to wells and incubated for 45 min at room temperature. Plates were washed again and 100 μl of horseradish peroxidase (HRP)-conjugated anti-horse immunoglobulin (Sigma) diluted 1:5000 was added to each well and incubated as described. Wells were washed a third time and 100 μl of *o*-phenylenediamine (Sigma) (1 mg/ml) plus 4 μl of 30% (v/v) hydrogen peroxide were added. Spectrophotometric determination of color change was recorded at 495 nm (A495nm) in a Multi-Mode Microplate Reader (Biotek). Anti-(*Bothrops jararaca* + *Crotalus durissus terrificus*) snake AV (FUNED, Belo Horizonte, Brasil) was included as a negative control. Data were fitted by five-parameter logistic regression implemented in GraphPad Prism 7 (GraphPad Software, San Diego, CA). AV dilutions corresponding to half-maximal A495nm values were calculated based on this software.

### Competitive solid-phase enzyme immunoassay

Inhibition of binding of anti-*T*. *trivitattus* (INPB, Argentina) antivenom to solid-phase-bound *T*. *trivittatus* venom from Argentina by competing with venoms from *T*. *trivittatus* (Paraguay) and *T*. *trivittatus* (Argentina) in solution was carried out according to the method of King et al. [21]. Briefly, antivenom samples, at a dilution corresponding to half the maximal binding to solid-phase antigens, were pre-incubated for 1 hour at room temperature with serially diluted venoms starting at 100 ng/ml. Then, 100 μl of the mixtures was allowed to bind to solid-phase venoms for 1 hour and the bound horse F(ab′)_2_s were detected as described in section 2.5. Values represent the highest inhibition of antibody binding to solid-phase venoms when high concentrations of competitor venoms in the liquid phase were used.

### Mass spectrometry of crude venoms

Mass spectra of positively charged ions from scorpion venoms were analyzed by MALDI–TOF MS in a Biflex III MALDI–TOF MS (Bruker, FRG). Samples for analyses (200–500 μg) were lyophilized, dissolved in 100 μL of ultra-pure water and diluted 10-fold with 0.1% (v/v) trifluoroacetic acid (TFA). A total of 1 μL of the diluted sample was mixed with 5 μL of matrix solution [10 mg/mL of 3,5-dimethoxy-4-hydroxycinnamic acid in a 1:1 mixture of acetonitrile and 0.1% TFA (v/v)]. One μL from this mixture was spotted on the target plate. Mass spectra of positively charged ions were recorded on a Autoflex III instrument operated in the linear mode. The total acceleration voltage and the detector voltage were 19 kV and 0.55 kV, respectively. A total of 100 to 150 single shots were accumulated for each sample. Masses were calculated from at least three independent analyses.

### Mass spectrometry identification of electrophoretic bands

Selected Coomassie-stained protein bands of the SDS-PAGE electrophoresed venoms were excised and in-gel digested overnight with sequencing-grade trypsin (Sigma), after reduction of disulfide bonds with DTT and alkylation with iodoacetamide, in an automated workstation (Intavis). The resulting peptides were submitted to nESIMS/MS on a Q-Exactive Plus mass spectrometer (Thermo). Twelve μL of each tryptic digest were loaded on a 2 cm×75 μm trap column, washed, and separated at 200 nL/min on a C18 Easy-spray analytical column (15 cm×75 μm, 3 μm particle) using a nano-Easy 1200 chromatograph. A gradient from 0.1% formic acid (solvent A) to 80% acetonitrile with 0.1% formic acid (solvent B) was developed as follows: 1–5% B in 1 min, 5–26% B in 25 min, 26–79% B in 4 min, 79–99% B in 1 min, and 99% B in 4 min, for a total of 35 min. MS spectra were acquired in positive mode at 2.0 kV, with a capillary temperature of 200°C, using 1 μscan at 400–1600 m/z, maximum injection time of 50 msec, AGC range of 1×10^6^, and resolution of 70,000. The top 10 ions with 2–5 positive charges were fragmented with an AGC target of 3×10^6^, minimum AGC 2×10^3^, maximum injection time 110 ms, dynamic exclusion time 5 s, and resolution 17,500. MS/MS spectra were searched for matches against protein sequences contained in the UniProt/SwissProt database (Arachnida, January 2020) using Peaks X software. Cysteine carbamidomethylation was set as a fixed modification, while deamidation of asparagine or glutamine and methionine oxidation were set as variable modifications, allowing up to 3 missed cleavages by trypsin. Parameters for match acceptance were set to FDR < 0.1%, -10lgP protein score ≥70, with at least 1 unique peptide.

### Determination of Proteolytic and Hyaluronidase activity by Substrate Zymography

To determine proteolytic activity and the molecular weight of proteases present in the venoms, gelatin zymography was performed according to [22]. Briefly, venom proteins (20 μg) were separated by SDS-PAGE in 20% gels containing type-A gelatin (Sigma) at a concentration of 0.25 mg/mL under non-reducing conditions. After washing for 1 h with 1% (v/v) Triton X-100 to remove SDS, gels were incubated at 37°C for 24 h in 50 mM Tris-HCl, pH 8.0, containing 5 mM CaCl_2_, and stained with Coomassie Blue R-250. Hyaluronidase activity present in the venoms (20 μg) was determined by the method reported by [23] based on SDS-PAGE in a 12% gel containing 0.5 mg/mL hyaluronic acid from rooster comb (Sigma). Incubation buffer (0.1 M NaCl, 0.1 M sodium phosphate) was adjusted to pH 6.6. Gels were stained with Alcian Blue 8GX (Sigma).

### DNA extraction and PCR amplification

Total DNA was extracted according to [24] from pedipalp muscle of two *T*. *trivittatus* specimens collected inside homes in Asunción, Paraguay (specimen 1 from Ciudad Nueva, 25.293631 S, 57.615761 W; specimen 2 from Barrio Jara, 25.274722 S, 57.603333 W). Scorpions were identified by David J. Guerrero, Natural History Museum, Asunción, based on [25,11]. Amplification and sequencing of the nucleotide sequence encoding the N-terminal portion of cytochrome oxidase subunit I (COI hereafter) was performed according to [26] using primers LCO1490: 5′-GGTCAACAAATCATAAAGATATTGG-3′ [27], and HCOEXTERNB: 5′-CCTATTGAWARAACATARTGAAAATG-3′ [28]. Amplified fragments were bidirectionally sequenced using an Applied Biosystems 3130 Genetic Analyzer DNA sequencer as previously described [24]. Sequences generated for this study were deposited at GenBank under the accession numbers MT800756 and MT808337.

### Phylogenetic analyses

A phylogeny and divergence dates among individuals were simultaneously estimated using Bayesian inference (BI) in BEAST 1.8.0 [29]. Consensus sequences were aligned in Geneious v. 7.1.7 (Biomatters Ltd., Auckland, New Zealand) using MUSCLE [30], checked for accuracy by eye, and trimmed to minimize missing characters. We determined the best-fit model of nucleotide substitution with MEGAX [31] using the Bayesian Information Criterion. We generated an.xml file in BEAUTi (BEAST package) using the best-fit substitution model (HKY+G), the uncorrelated lognormal clock model, and the Yule tree prior. Preliminary BEAST runs using the uncorrelated lognormal clock model revealed a low ucld.stdev value (<1.0), so we used a strict clock model for final runs (as suggested in the BEAST manual).To calibrate the BEAST analyses, we used normal clock rate priors with a mean rate (ucld.mean) of 0.007 substitutions per site per million years, as previously estimated for other buthid scorpions [32]. Following [33], we adjusted the standard deviation so 95% of the normal distribution included minimum and maximum rates estimated for COI in other studies of scorpions (SD: 0.00270). We conducted two independent MCMC runs for 20 million generations each and sampled every 10,000 generations. Tracer 1.6 was used to confirm adequate effective sample sizes and that Markov chains reached stationarity and convergence. The runs were combined to produce a maximum clade credibility tree using Treeannotator (BEAST package) and visualized in Figtree 1.4.0 (http://tree.bio.ed.ac.uk/software/).

### Statistical analyses

The Spearman-Karber method [15] was used to determine venom lethality. ELISA curves were analyzed by non-linear regression and half-maximal A490nm values (including their 95% confidence intervals) calculated using the software Prism7.0 (GraphPad Inc., CA). The significance of statistical differences between half-maximal values corresponding to AV recognition of *T*. *trivittatus* venoms from Argentina and Paraguay was evaluated using the Extra sum-of-squares F test implemented in Prism7.0 (*p* < 0.05).

## Results

### Lethality of *T*. *trivittatus* venom from Paraguay

Medium lethal dose in NIH Swiss mice intraperitoneally injected with *T*. *trivittatus* venom (a pool from female and male specimens) was estimated as 1.19 mg/kg (95% CI: 0.89–1.71). Mice injected with doses as low as 0.74 mg/kg presented with signs of acute toxicity such as profuse salivation, piloerection, urination, voiding of feces, extension rigidity of the hindlimbs, and dyspnea starting 10 minutes after venom administration. At lower doses (0.74 and 1.10 mg/kg) manifestations subsided after 45 minutes. When doses were lethal, death was usually recorded 45–60 min post-injection. In some mice injected with lethal doses (1.66 and 2.48 mg/kg) mouth bleeding was observed.

### *In vivo* neutralization of *T*. *trivittatus* venom from Paraguay by therapeutic scorpion antivenoms

To test the neutralizing capacity of therapeutic anti-*Tityus* antivenoms towards venom from the Paraguayan population of *T*. *trivittatus*, Swiss mice were injected i.p. with 0.2 mL amounting to 3×LD_50_s pre-incubated with 100 μL of antivenoms produced in Brazil, and Argentina. Table 1 summarizes representative survival data from two independent experiments, including a negative control, using snake AV (anti-*B*. *jararaca* + *C*. *durissus terrificus*), and a positive control, comprising mice injected with 3×LD_50_s in the presence of PBS. The Brazilian (Instituto Vital Brazil) anti-*T*. *serrulatus* showed the highest protection (100% survival), followed by the Argentinean anti-*T*. *trivittatus* (INPB thereinafter) AV (50% survival). Mice injected with mixtures of venom and Brazilian AV showed no symptoms of toxicity. Mice injected with mixtures of venom and Argentinean AV presented with toxicity signs such as dyspnea, salivation and piloerection after 10 minutes, which subsided 1 hour post-injection in the case of surviving animals. Protein concentrations of tested AVs were 28.1 ± 0.9 mg/mL (Brazilian AV) and 33.1 ± 2.8 mg/mL (INPB AV).

**Table 1:** Neutralization of *Tityus trivittatus* (Paraguay) venom by therapeutic AVs^a^.

Treatment	LD_50_	Surviving mice /total mice	Survival percentage
Phosphate buffer saline	0	4/4	100
Phosphate buffer saline	3	0/4	0
Snake AV (Funed, Brazil)	3	0/4	0
Anti-*Tityus serrulatus* AV (Instituto Vital Brazil, Brazil)	3	4/4	100
Anti-*Tityus trivittatus* AV (INPB, Argentina)	3	2/4	50

a Data are representative of two experiments conducted independently.

### SDS-PAGE and cross-recognition by immunoblotting of *Tityus* spp. venoms using therapeutic antivenoms

**Fig 2:**
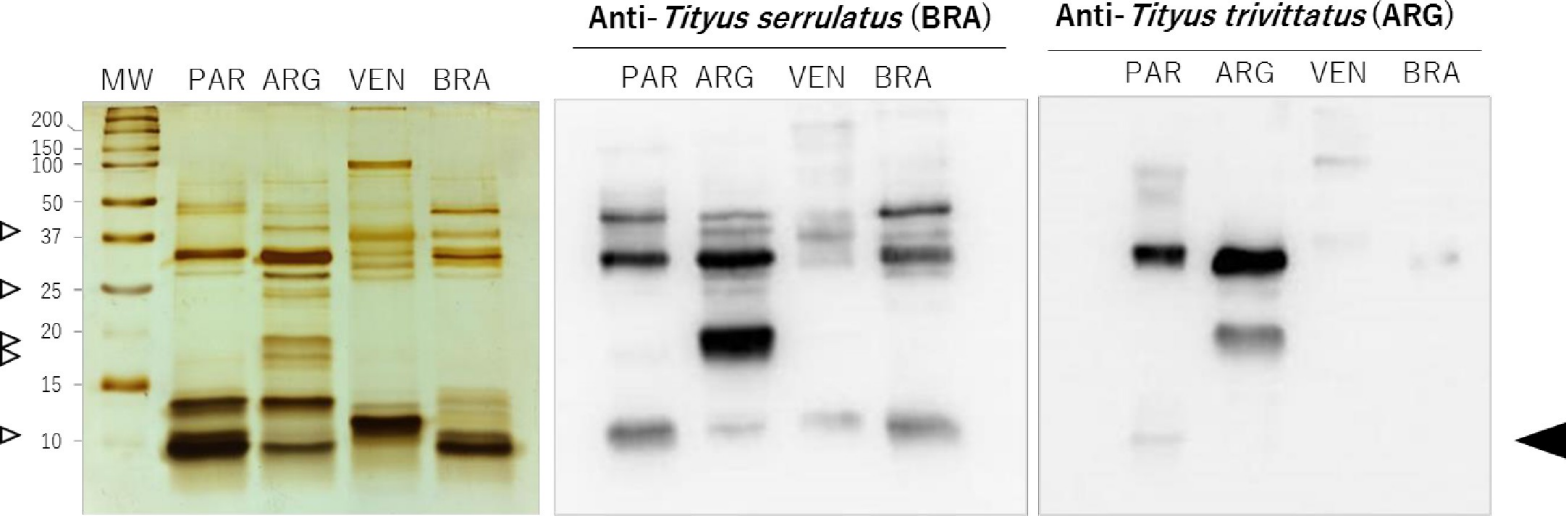
Cross-recognition by immunoblotting of *Tityus* spp. venoms using therapeutic AVs. (Left panel) Silver-stained SDS-PAGE (20% gel) under reducing conditions of *Tityus* ssp. venoms from Paraguay (PAR, *T*. *trivitattus*), Argentina (ARG, *T*. *trivittatus*), Venezuela (VEN, *T*. *discrepans*), and Brazil (BRA, *T*. *serrulatus*). (Right panels) Blots developed with therapeutic horse sera from Brazil and Argentina. Arrows on the left indicate migration of *T*. *trivittatus* population-specific components. Arrow on the right indicates migration of low molecular mass scorpion toxins. MW, molecular mass markers.

Fig 2 shows the result of immunoblottings performed to identify *T*. *serrulatus* and *T*. *trivittatus* (Argentina and Paraguay) venom protein components recognized by Brazilian and Argentinean scorpion AVs. Venom from Venezuelan *T*. *discrepans* was included as an additional control considering its reported low recognition by the Brazilian AV and its phylogenetic separation from southern South American *Tityus* spp. including *T*. *serrulatus* and *T*. *trivittatus* [1]. Blots developed with the Brazilian AV showed a greater number of recognized components in *T*. *serrulatus* and *T*. *trivittatus* (both populations) compared to the INPB AV, particularly the low molecular mass fraction (<10 kDa) which corresponds to scorpion toxins targeting ion channels [34]. This fraction was only weakly detected using Argentinean antibodies. Most intensely recognized protein bands corresponded to high molecular mass components in both AVs tested (17–60 kDa). The INPB AV recognized a band of 30 kDa and a faint signal corresponding to the low mass neurotoxic fraction in Paraguayan samples, whereas the 30 kDa component was more intensely detected and an additional band of 20 kDa was recognized in the case of the Argentinean population. *T*. *discrepans* venom components were only weakly recognized by both AVs. Banding patterns in silver-stained SDS-PAGE gels differed between *T*. *trivittatus* venoms from Paraguay and Argentina, notably with proteins of 17, 19, 25, and 40 kDa being only present in the Argentinean venom, together with an intense 30-kDa component, and a fraction migrating around 10 kDa only found in the Paraguayan population (Fig 2, left panel).

### *T*. *trivitattus* venom antigenicity evaluated by ELISA

**Fig 3:**
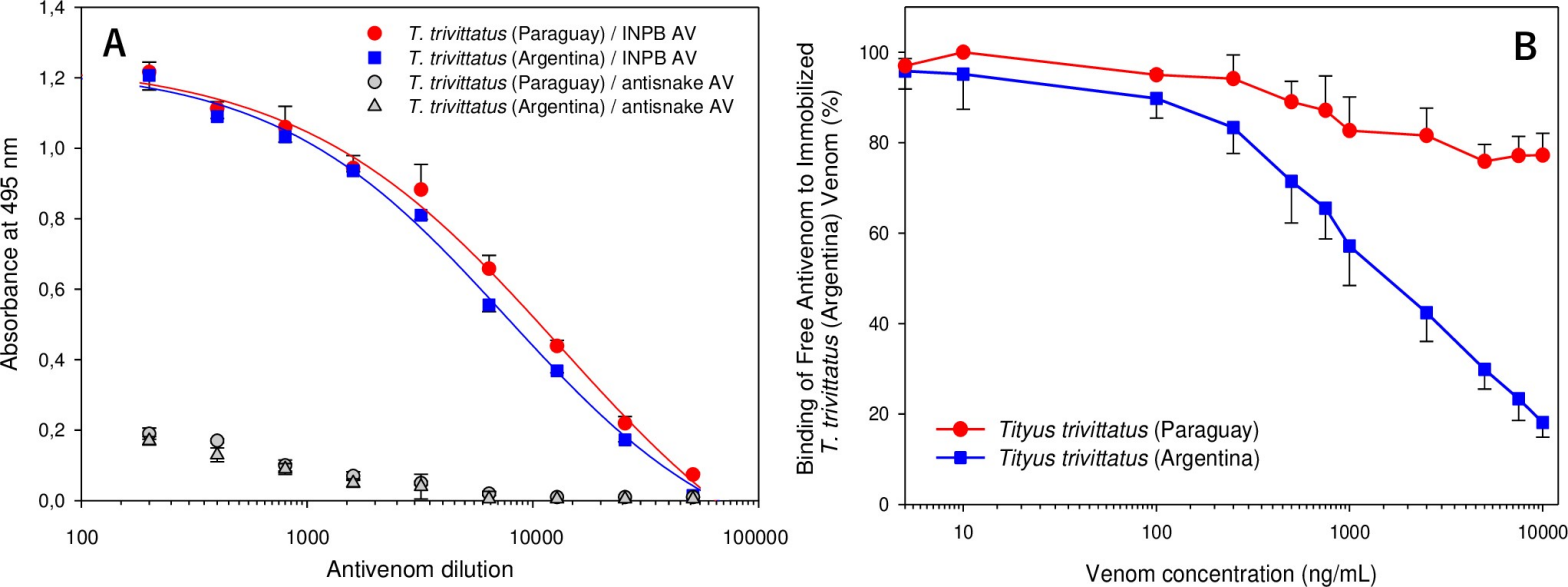
Antigenicity of *T*. *trivittatus* venom populations. ELISA titration for venom reactivity towards INPB horse antibodies. Reactivity towards a snake antivenom (anti-*B*. *jararaca* + *C*. *durissus terrificus*) was used as negative control (Panel A). Competitive ELISA using *T*. *trivittatus* (Argentina) as control venom to evaluate inhibition of therapeutic antivenom binding with competing *T*. *trivittatus* venoms from Argentina and Paraguay (Panel B). Results are representative of three experiments conducted independently. Bars at each value represent mean ± standard error of the mean.

Considering the differences in electrophoretic composition and *in vivo* and *in vitro* reactivity towards the INPB AV between venoms from Paraguayan and Argentinean *T*. *trivittatus* populations, ELISA tests were performed to compare their antigenicity. Particularly, we wanted to investigate quantitatively antigenic differences between these venoms as indicated by immunoblotting. Fig 3 shows titration of AV reactivity towards venoms of both populations (Panel A) (Data available in S1 Data). To estimate the statistical significance of differences between recognition of both venoms by the INPB AV, half-maximal AV dilution values were compared by the Extra Sum-of-Squares F-test implemented in GraphPad Prism4. A comparison of these values (Paraguay: 4.16 ± 0.15, 95%CI: 3.95–5.01; Argentina: 3.97 ± 0.06, 95%CI: 3.86–4.16) rendered the difference nonsignificant (F = 1.71, *p* = 0.198), implying that *T*. *trivittatus* venoms from Paraguay and Argentina were similarly recognized by the anti-*T*. *trivittatus* (INPB) AV. Panel B shows the results of a competitive ELISA assay for testing the inhibition capacity of *T*. *trivittatus* venoms from Paraguay and Argentina on the binding of INPB horse antibodies to immobilized *T*. *trivittatus* (Argentina) venom (Data available in S2 Data). Whereas venom from Argentina produced 18.1 ± 3.3% free antibodies at the maximal venom dose tested (10 μg/mL), incubation with venom from Paraguay produced 77.2 ± 4.2% free antibodies at the same concentration.

### Enzyme activity comparison between *T*. *trivittatus* venoms from Paraguay and Argentina by Zymography

**Fig 4:**
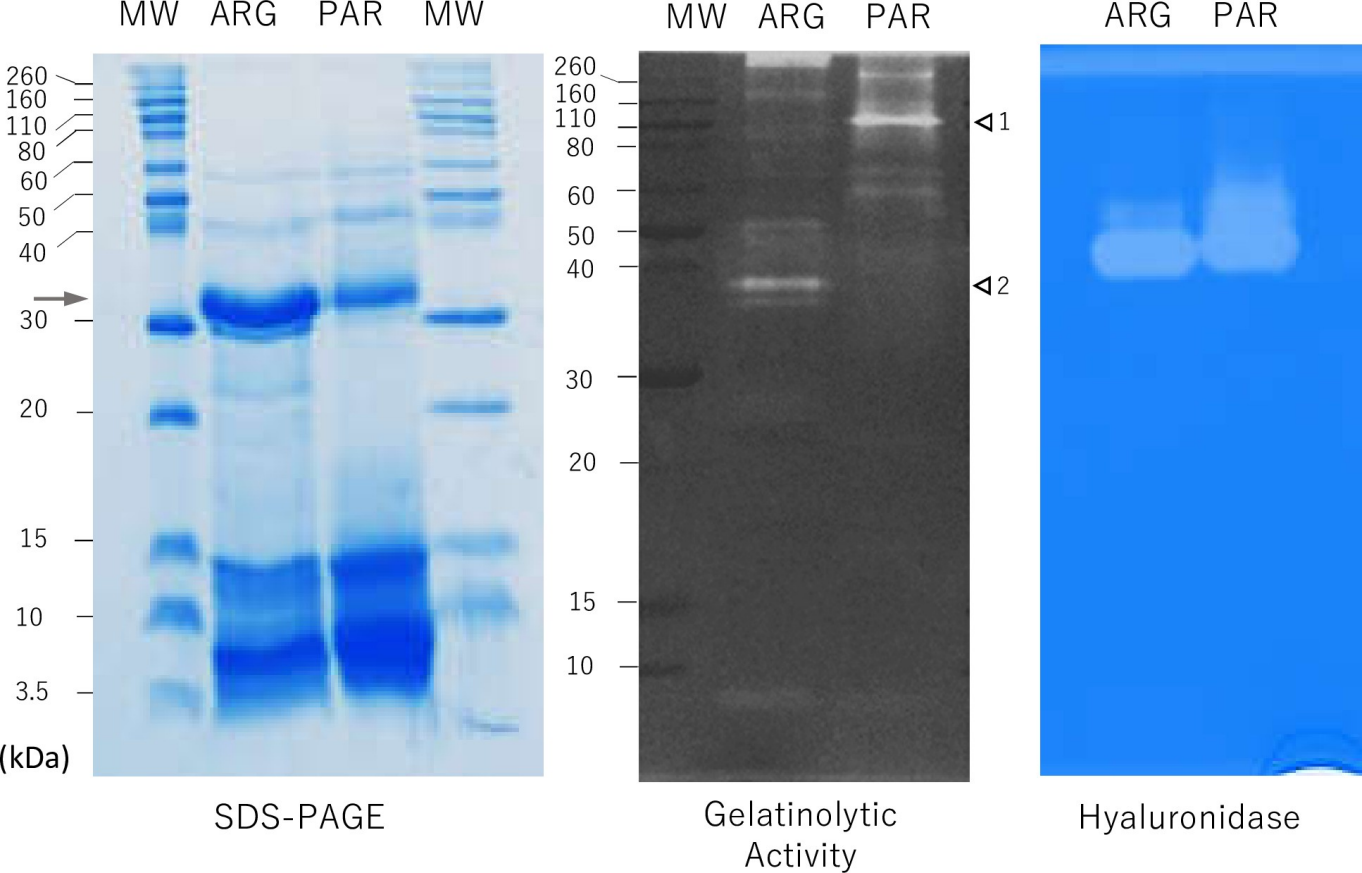
Proteolytic (gelatinolytic) and hyaluronic acid-degrading activities of *T*. *trivittatus* venoms. (Left panel) Coomassie blue-stained SDS PAGE (20% gel) of *T*. *trivittatus* venoms from Argentina and Paraguay electrophoresed under non-reducing conditions. Arrow indicates bands excised for proteomic identification in both venoms (see section 3.7 for details). (Middle panel) Protein components with gelatin-degrading activity. Arrows indicate major bands with gelatinolytic activity in venoms from Paraguay (**1**, 110 kDa) and Argentina (**2**, 37 kDa). (Right panel) Protein components with hyaluronidase activity identified after venom separation in the presence of hyaluronic acid. MW = molecular mass markers.

Fig 4 shows the results of the evaluation of in-gel enzyme activity by substrate zymography in *T*. *trivittatus* venoms. In the presence of hyaluronic acid as a substrate, we detected hyaluronidase activity at 40–50 kDa in both venoms. In the presence of gelatin, several bands with proteolytic activity were identified, which were distinct between *T*. *trivittatus* populations. The main proteolytic component unique to the Argentinean population venom migrated at 37 kDa, whereas the main component of the Paraguayan population was close to 110 kDa. Other population-specific, higher molecular mass minor components were also evident in both zymograms.

### MALDI-TOF mass spectrometry assessment of *T*. *trivittatus* (Paraguay and Argentina) venom composition

**Fig 5:**
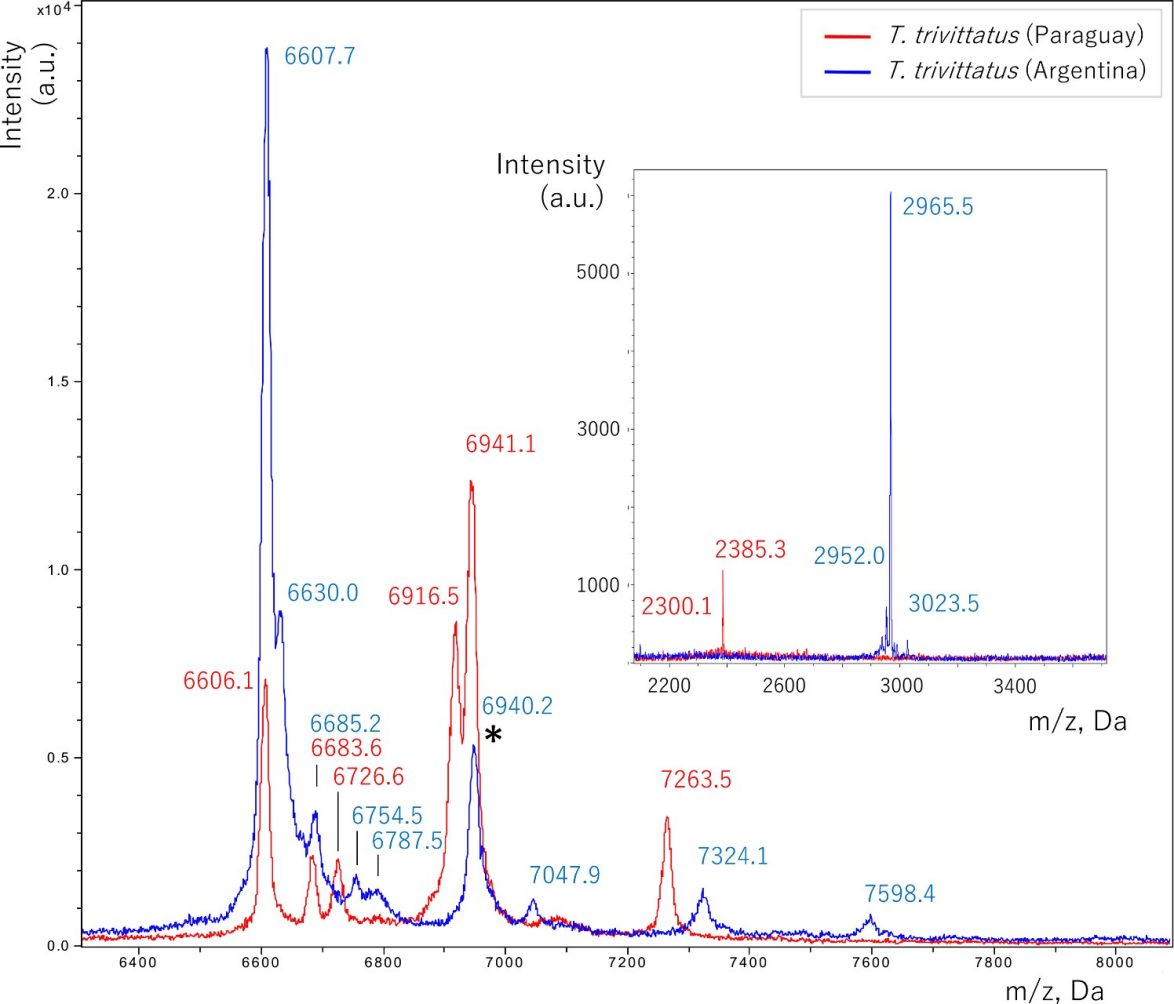
Overlapped MALDI-TOF MS spectra of *T*. *trivittatus* venoms from Paraguay and Argentina in the 6400–8000 (main Fig) and 2000–3500 (inset) Da m/z ranges. Components in either spectra are labeled with their corresponding *m/z* value (Table 2). (*) identifies a component with molecular mass matching that corresponding to *T*. *trivittatus* (Argentina) NaTx toxin Tt1g [7].

Fig 5 shows spectra obtained through MALDI-TOF to compare protein mass distributions in venoms from the two *T*. *trivitattus* populations. Table 2 presents a list of the main ions observed in both venoms by MALDI-TOF. In the NaTx mass range (6–8 kDa), peptides unique to the Paraguayan population were components with m/z 6726.6, 6916.5, and 7263.5 Da. Whereas peptides unique to the Argentinean population were 6630.0, 6754.5, 6787.5, 7047.9, 7324.1, and 7598.4 Da. Both populations shared two components in this mass range (± 2 Da), 6606.1 and 6941.1. No components were shared in the mass range of KTx and antimicrobial peptides (2–5 kDa) [34] (see inset Fig 5 and Table 2).

**Table 2:** Main ions (monoprotonated m/z vales) observed in *T*. *trivittatus* venoms by MALDI-TOF within the m/z range 2000–12,000. PAR, Paraguay; ARG, Argentina.

*T*. *trivittatus* PAR	*T*. *trivittatus* ARG
2300.1	2952.0
2385.3	2965.5
5315.7	3023.5
6606.1	5519.9
6683.6	6607.7
6726.6	6630.0
6916.5	6685.2
6941.1	6754.5
7263.5	6787.5
10755.8	6940.2
10951.4	7047.9
	7324.1
	7598.4
	9358.9
	9828.9
	10447.7
	10959.114
	10978.959

### Proteomic identification of proteins in 30-kDa SDS PAGE bands from *T*. *trivittatus* venoms

A major component of venoms from both *T*. *trivittatus* populations is a protein of molecular mass *ca*. 30 kDa, which appears at a higher abundance in the Argentinean samples (Figs 2 and 4). Therefore, we digested bands excised from SDS-PAGE gels (Fig 4) with trypsin and analyzed the resulting peptides by nanoelectrospray ionization tandem MS/MS (nESIMS/MS). Tables 3 and 4 summarize the identified venom peptides. Significant sequence matches were found for peptides from both populations with putative venom metalloproteinases from *T*. *bahiensis* (identified through transcriptomics [35]), metalloserrulases from *T*. *serrulatus* (which are metalloproteases identified both at the molecular and functional levels [36,37]), and a hyaluronidase from *T*. *bahiensis* (UniProtKB A0A0C9RFM5) (identified at the transcript level [35]). Both *T*. *trivittatus* venoms shared all listed *T*. *bahiensis* putative proteases and *T*. *serrulatus* metalloserrulases 18 (UniProtKB A0A1S5QN52) and 20 (UniProtKB A0A1S5QN67). Peptides matching *T*. *serrulatus* metalloserrulases 1 (UniProtKB A0A076L876) and 16 (UniProtKB A0A1S5QN57) (from *T*. *serrulatus*) were only found in the venom from Paraguay (Table 3).

**Table 3:** Protein matches obtained by nESI-MS/MS of tryptic peptides from the SDS-PAGE 30 kDa band of *Tityus trivittatus* (Paraguay) venom^a^^,^^b^. Accession codes in bold correspond to protein matches unique to the Paraguayan *T*. *trivittatus* population.

Accession	-10lgP	% Cov	#Pept	#Unique	Avg. mass	m/z	z	Matching peptide sequences^a^	Description
A0A0C9QKU3	221.51	32	24	12	44081	484.2586 665.6638 577.7932 569.2990 473.7247 487.7671 499.7458 479.9159 681.8162 593.6390 526.9367 571.7819 412.2187	3 3 2 2 2 2 4 3 2 3 3 4 3	K.YVHSDIIYKANK.Y K.ESDVQNGKYVHSDIIYK.A K.ESDVQNGKYVHSDIIYKANK.Y K.YVHSDIIYK.A K.ANKYYC(+57.02)K.N Y.VHSDIIYK.A K.ESDVQN(+.98)GKYVHSDIIYK.A Q.N(+.98)GKYVHSDIIYK.A K.ESDVQNGKYVHS.D S.DVQNGKYVHSDIIYK.A S.DIIYKANKYYC(+57.02)K.N K.ENEPSYIKESDVQNGKYVHS.D Y.DTM(+15.99)NLDIKIR.L	Putative metalloproteinase, TSA: *Tityus bahiensis* Tbah00944 mRNA sequence (Fragment)
A0A0C9S3A4	185.28	23	15	14	43397	734.3187 612.3328 401.2473 451.2544 748.8595 504.9067 597.5231 601.5221 596.6284 586.2556 783.8533 448.1848 502.2550 632.7864 841.3672 466.6990	2 2 3 3 2 3 4 4 3 2 2 2 2 4 2 2	K.VGVAQDDSDYNER.V K.C(+57.02)VEHLLSLPR.A K.AQVIGITPFKK.V K.KC(+57.02)VEHLLSLPR.A N.DGYIMGSGNNKVNK.F N.DGYIM(+15.99)GSGNNKVNK.F K.DC(+57.02)PENDGYIMGSGNNKVNKFK.F K.DC(+57.02)PENDGYIM(+15.99)GSGNNKVNKFK.F N.DGYIM(+15.99)GSGNNKVNKFK.F N.DGYIM(+15.99)GSGNNK.V K.VGVAQDDSDYNERV.D R.ASC(+57.02)VLADC(+57.02) E.ETGLSGSPGAK.D K.VGVAQDDSDYNERVDTVAHETAH.L K.VGVAQDDSDYNERVD.T K.YYC(+57.02)NNAK.G	Putative metalloproteinase, TSA: *Tityus bahiensis* Tbah00729 mRNA sequence (Fragment)
A0A0C9RFK9	183.09	20	11	11	44944	470.9696 500.2694 422.5527 663.2826 404.2213 522.2686 503.2654 454.5795 535.2839 498.7635 404.5492 545.7296	4 4 3 2 3 3 3 3 2 2 3 2	K.SHTFC(+57.02)TPSTC(+57.02)KIEAGGK.V K.VTESDKKTILDTHNQLR.N K.LASGKENQYQK.L K.SHTFC(+57.02)TPSTC(+57.02)K.I K.TILDTHNQLR.N K.VGC(+57.02)GVAGYVENGVKR.V R.NKLASGKENQYQK.L K.IEAGGKVTESDKK.T K.SVTPDGPQIR.R I.LDTHNQLR.N K.TILDTHN(+.98)QLR.N K.FEHDSGDQR.A	Putative metalloproteinase, TSA: *Tityus bahiensis* Tbah00905 mRNA sequence
A0A0C9QKW3	169.79	17	12	8	42480	686.5908 549.6150 776.6194 780.6169 617.3062 663.6675 492.2530 498.2787 560.2844	4 3 4 4 2 3 3 2 2	D.PREDGTVDINTAGIANSAGVC(+57.02)KPC(+57.02)LK.A N.TAGIANSAGVC(+57.02)KPC(+57.02)LK.A R.GMGDPREDGTVDINTAGIANSAGVC(+57.02)KPC(+57.02)LK.A R.GM(+15.99)GDPREDGTVDINTAGIANSAGVC(+57.02)KPC(+57.02)LK.A A.NSAGVC(+57.02)KPC(+57.02)LK.A V.DINTAGIANSAGVC(+57.02)KPC(+57.02)LK.A A.GIANSAGVC(+57.02)KPC(+57.02)LK.A R.FKSNSALTK.Y N.SAGVC(+57.02)KPC(+57.02)LK.A	Putative metalloproteinase, TSA: *Tityus bahiensis* Tbah00248 mRNA sequence (Fragment)
A0A218QX25	163.62	17	12	1	45537	833.8954 833.6491	4 4	R.TITIAHEAGHM(+15.99)LGVPHDGHESTEVGVPN(+.98)GPGAK.S R.TITIAHEAGHM(+15.99)LGVPHDGHESTEVGVPNGPGAK.S	Putative metalloproteinase (Fragment) *Tityus serrulatus*
A0A218QX15	162.04	17	11	1	44723	830.9042 831.1525	4 4	R.TITIAHEAGHMLGLPHDGQESTEVGVPNGPGAK.S R.TITIAHEAGHMLGLPHDGQESTEVGVPN(+.98)GPGAK.S	Putative metalloproteinase *Tityus serrulatus*
A0A218QXI9	147.71	14	9	9	37523	831.8368 540.7810 442.5711 597.5912 655.3565 823.8421 702.2933 527.2145 629.8230 427.2144 440.7540	2 4 3 3 2 2 3 2 2 2 2	K.EGYIM(+15.99)GNDYGENER.K R.KFSPC(+57.02)SKANIM(+15.99)YFLGKPR.A K.ANIM(+15.99)YFLGKPR.A K.EGYIM(+15.99)GNDYGENERK.F K.ANIMYFLGKPR.A K.EGYIMGNDYGENER.K K.C(+57.02)PGKEGYIM(+15.99)GNDYGENER.K M.GNDYGENER.K R.AIGEPRPDGTVF.D R.KFSPC(+57.02)SK.A M.YFLGKPR.A	Putative metalloproteinase (Fragment) *Tityus serrulatus*
A0A1S5QN52	147.71	12	9	9	43885	831.8368 540.7810 442.5711 597.5912 655.3565 823.8421 702.2933 527.2145 629.8230 427.2144 440.7540	2 4 3 3 2 2 3 2 2 2 2	K.EGYIM(+15.99)GNDYGENER.K R.KFSPC(+57.02)SKANIM(+15.99)YFLGKPR.A K.ANIM(+15.99)YFLGKPR.A K.EGYIM(+15.99)GNDYGENERK.F K.ANIMYFLGKPR.A K.EGYIMGNDYGENER.K K.C(+57.02)PGKEGYIM(+15.99)GNDYGENER.K M.GNDYGENER.K R.AIGEPRPDGTVF.D R.KFSPC(+57.02)SK.A M.YFLGKPR.A	Metalloserrulase 18 *Tityus serrulatus*
A0A0C9RPA3	109.85	17	6	3	43099	724.6942 548.5833 487.1904	3 3 2	R.TMTQ(+.98)N(+.98)KPSGVVNAAGLAYYGK.V K.VC(+57.02)DEC(+57.02)YKVGATVDK.S K.VC(+57.02)DEC(+57.02)YK.V	Putative metalloproteinase, TSA: *Tityus bahiensis* Tbah01003 mRNA sequence
A0A0C9RP91	107.95	10	3	3	29546	492.9221 549.9507 418.2052	3 3 2	R.LGTVDRQSGPQYR.F L.KLTSPVDFDENINR.I R.QSGPQYR.F	Putative metalloproteinase, TSA: *Tityus bahiensis* Tbah01461 mRNA sequence (Fragment)
**A0A076L876**	105.83	9	5	1	42691	491.2649	2	Y.IVTDSAFTK.R	**Metalloserrulase 1 *Tityus serrulatus***
A0A1S5QN67	95.13	10	4	1	41560	487.7220	2	R.SFGNYVC(+57.02)K.N	Metalloserrulase 20 (Fragment) *Tityus serrulatus*
A0A0C9RFM5	90.92	10	5	5	46533	650.2638 482.2693 413.2194 509.7628 439.2125	2 2 2 2 2	K.DEPSQFSC(+57.02)SSR.I K.M(+15.99)PVFKPTK.I K.ITSDYVK.N K.VAKEEWEK.S R.IQMENSR.L	Hyaluronidase *Tityus bahiensis*
A0A218QXX3	71.45	5	2	2	37728	592.7829 452.7415	2 2	K.FSTC(+57.02)SVENIK.Y K.SDPPFITK.S	Putative metalloproteinase *Tityus serrulatus*
A0A218QXF3	71.45	5	2	2	42121	592.7829 452.7415	2 2	K.FSTC(+57.02)SVENIK.Y K.SDPPFITK.S	Putative metalloproteinase (Fragment) *Tityus serrulatus*
**A0A1S5QN57**	71.45	4	2	2	44932	592.7829 452.7415	2 2	K.FSTC(+57.02)SVENIK.Y K.SDPPFITK.S	**Metalloserrulase 16 *Tityus serrulatus***

a Peptide spectral matching search performed against the Uniprot Arachnida database, using Peaks X software

^b^m/z and z values correspond to listed peptide sequences.

**Table 4:** Protein matches obtained by nESI-MS/MS of tryptic peptides from the SDS-PAGE 30 kDa band of *Tityus trivittatus* (Argentina) venom^a^^,^^b^.

Accession	-10lgP	% Cov	#Pept	#Unique	Avg. mass	m/z	z	Matching peptide sequences	Description
A0A0C9QKU3	250.12	32	51	20	44081	838.4091 1122.872 499.4995 1117.8669 725.8853 1117.8693 569.2993 713.5822 577.7931 843.3818 1123.2007 628.8071 785.8694 473.7244 470.2470 585.2924 487.7671 526.9368 786.3618 499.7454 730.3516 693.3278 786.3599 842.6499 717.5781 692.6572 681.8154 629.2960 470.7375 482.7752 838.9047 633.2950 717.8291 1123.5376	4 3 4 3 2 3 2 4 4 2 3 2 2 2 2 2 2 3 2 4 3 2 2 4 4 3 2 4 2 2 4 4 4 3	R.TITIAHEAGHMLGVPHDGQESTEAEVPNGPGAK.S R.TITIAHEAGHM(+15.99)LGVPHDGQESTEAEVPNGPGAK.S K.ESDVQNGKYVHSDIIYK.A R.TITIAHEAGHMLGVPHDGQ(+.98)ESTEAEVPNGPGAK.S K.YVHSDIIYKANK.Y R.TITIAHEAGHMLGVPHDGQESTEAEVPN(+.98)GPGAK.S K.YVHSDIIYK.A A.HEAGHMLGVPHDGQESTEAEVPNGPGAK.S K.ESDVQNGKYVHSDIIYKANK.Y H.DGQESTEAEVPNGPGAK.S R.TITIAHEAGHM(+15.99)LGVPHDGQ(+.98)ESTEAEVPNGPGAK.S E.STEAEVPNGPGAK.S D.GQESTEAEVPNGPGAK.S K.ANKYYC(+57.02)K.N E.AEVPNGPGAK.S S.TEAEVPNGPGAK.S Y.VHSDIIYK.A S.DIIYKANKYYC(+57.02)K.N D.GQ(+.98)ESTEAEVPNGPGAK.S K.ESDVQN(+.98)GKYVHSDIIYK.A M.LGVPHDGQESTEAEVPNGPGAK.S Q.ESTEAEVPNGPGAK.S D.GQESTEAEVPN(+.98)GPGAK.S R.TITIAHEAGHM(+15.99)LGVPHDGQESTEAEVPN(+.98)GPGAK.S A.HEAGHM(+15.99)LGVPHDGQESTEAEVPNGPGAK.S L.GVPHDGQESTEAEVPNGPGAK.S K.ESDVQNGKYVHS.D A.GHMLGVPHDGQESTEAEVPNGPGAK.S E.AEVPN(+.98)GPGAK.S S.DIIYKANK.Y R.TITIAHEAGHMLGVPHDGQ(+.98)ESTEAEVPN(+.98)GPGAK.S A.GHM(+15.99)LGVPHDGQESTEAEVPNGPGAK.S A.HEAGHM(+15.99)LGVPHDGQ(+.98)ESTEAEVPNGPGAK.S R.TITIAHEAGHM(+15.99)LGVPHDGQ(+.98)ESTEAEVPN(+.98)GPGAK.S	Putative metalloproteinase, TSA: *Tityus bahiensis* Tbah00944 mRNA sequence (Fragment) OS = *Tityus bahiensis* OX = 50343 PE = 2 SV = 1
A0A218QX15	204.73	26	29	2	44723	834.9000 550.2748	4 2	R.TITIAHEAGHM(+15.99)LGLPHDGQESTEVGVPNGPGAK.S N.AVGIALGASAC(+57.02)NKC(+57.02)EK.V	Putative metalloproteinase OS = *Tityus serrulatus* OX = 6887 P E = 4 SV = 1
A0A218QX25	203.56	21	28	1	45537	829.6457 829.8973 1111.5361	4 4 3	R.TITIAHEAGHMLGVPHDGHESTEVGVPNGPGAK.S R.TITIAHEAGHMLGVPHDGHESTEVGVPN(+.98)GPGAK.S R.TITIAHEAGHM(+15.99)LGVPHDGHESTEVGVPN(+.98)GPGAK.S	Putative metalloproteinase (Fragment) OS = *Tityus serrulatus* OX = 6887 PE = 4 SV = 1
A0A0C9RFM9	186.62	31	21	1	21496	427.7211	2	A.DVPNGPGAK.S	Putative metalloproteinase, TSA: *Tityus bahiensis* Tbah02152 mRNA sequence (Fragment) OS = *Tityus bahiensis* OX = 50343 PE = 2 SV = 1
A0A0C9S3A4	178.48	23	16	16	43397	734.3187 612.3304 688.8055 597.5222 499.5753 586.2545 601.3669 451.2536 434.5468 704.6401 502.2548 504.9069 578.2572 783.8535 448.1844 466.6985 467.1908 709.9709 467.1908 601.5212 596.6279 778.8513	2 2 2 4 3 2 2 3 3 3 2 3 2 2 2 2 2 3 2 4 3 4	K.VGVAQDDSDYNER.V K.C(+57.02)VEHLLSLPR.A K.NMAKYYC(+57.02)NNAK.G K.DC(+57.02)PENDGYIMGSGNNKVNKFK.F N.DGYIMGSGNNKVNK.F N.DGYIM(+15.99)GSGNNK.V K.AQVIGITPFKK.V K.KC(+57.02)VEHLLSLPR.A K.YYC(+57.02)NNAKGLAK.D K.DC(+57.02)PENDGYIMGSGNNKVNK.F E.ETGLSGSPGAK.D N.DGYIM(+15.99)GSGNNKVNK.F N.DGYIMGSGNNK.V K.VGVAQDDSDYNERV.D R.ASC(+57.02)VLADC(+57.02) K.YYC(+57.02)NNAK.G K.YYC(+57.02)NN(+.98)AK.G K.DC(+57.02)PENDGYIM(+15.99)GSGNNKVNK.F K.YYC(+57.02)N(+.98)NAK.G K.DC(+57.02)PENDGYIM(+15.99)GSGNNKVNKFK.F N.DGYIM(+15.99)GSGNNKVNKFK.F E.ETGLSGSPGAKDC(+57.02)PENDGYIM(+15.99)GSGNNKVNK.F	Putative metalloproteinase, TSA: *Tityus bahiensis* Tbah00729 mRNA sequence (Fragment) OS = *Tityus bahiensis* OX = 50343 PE = 2 SV = 1
A0A0C9RFK9	176.57	27	15	15	44944	470.9690 663.2826 422.5525 500.2693 404.2212 503.2651 454.5787 522.2667 514.2599 535.2840 545.7285 498.7633 540.7415 479.7355 412.8637	4 2 3 4 3 3 3 3 3 2 2 2 2 4 3	K.SHTFC(+57.02)TPSTC(+57.02)KIEAGGK.V K.SHTFC(+57.02)TPSTC(+57.02)K.I K.LASGKENQYQK.L K.VTESDKKTILDTHNQLR.N K.TILDTHNQLR.N R.NKLASGKENQYQK.L K.IEAGGKVTESDKK.T K.VGC(+57.02)GVAGYVENGVKR.V K.DVTMTGSKPFTTQK.V K.SVTPDGPQIR.R K.FEHDSGDQR.A I.LDTHNQLR.N P.ENC(+57.02)PEIYR.R K.AC(+57.02)KDVTM(+15.99)TGSKPFTTQK.V P.ENC(+57.02)PEIYRR.L	Putative metalloproteinase, TSA: *Tityus bahiensis* Tbah00905 mRNA sequence OS = *Tityus bahiensis* OX = 50343 PE = 2 SV = 1
A0A218QWW8	156.90	24	18	1	34355	607.9504	3	K.EN(+.98)EPSFIKESDVQNGK.Y	Putative metalloproteinase (Fragment) OS = *Tityus serrulatus* OX = 6887 PE = 4 SV = 1
A0A0C9QKW7	103.50	20	9	1	25764	656.9733	3	I.HN(+.98)AN(+.98)NYYC(+57.02)KNATGLAQK.A	Putative metalloproteinase, TSA: *Tityus bahiensis* Tbah00001 mRNA sequence (Fragment) OS = *Tityus bahiensis* OX = 50343 PE = 2 SV = 1
A0A0C9QKW3	98.80	15	5	5	42480	515.2409 441.2169 515.7537 449.2143 434.2216 780.6211	2 2 2 2 2 4	E.GSPGAANC(+57.02)PAK.A K.AGYIMGNR.N K.YKFSPC(+57.02)TK.K K.AGYIM(+15.99)GNR.N K.FSPC(+57.02)TKK.C R.GM(+15.99)GDPREDGTVDINTAGIANSAGVC(+57.02)KPC(+57.02)LK.A	Putative metalloproteinase, TSA: *Tityus bahiensis* Tbah00248 mRNA sequence (Fragment) OS = Tityus bahiensis OX = 50343 PE = 2 SV = 1
A0A0C9RFM5	97.68	13	7	6	46533	474.2709 650.2643 482.2698 439.2124 513.5931 470.8954 509.7636	2 2 2 2 3 3 2	K.MPVFKPTK.I K.DEPSQFSC(+57.02)SSR.I K.M(+15.99)PVFKPTK.I R.IQMENSR.L S.KHQEWPSDRVEK.V K.HQEWPSDRVEK.V K.VAKEEWEK.S	Hyaluronidase OS = *Tityus bahiensis* OX = 50343 PE = 2 SV = 1
A0A218QXI9	95.40	11	5	4	37523	442.5718 831.8405 696.9644 554.9958	3 2 3 4	K.ANIM(+15.99)YFLGKPR.A K.EGYIM(+15.99)GNDYGENER.K K.C(+57.02)PGKEGYIMGNDYGENER.K K.C(+57.02)PGKEGYIMGNDYGENERK.F	Putative metalloproteinase (Fragment) OS = *Tityus serrulatus* OX = 6887 PE = 4 SV = 1
A0A1S5QN52	95.40	9	5	4	43885	442.5718 831.8405 696.9644 554.9958	3 2 3 4	K.ANIM(+15.99)YFLGKPR.A K.EGYIM(+15.99)GNDYGENER.K K.C(+57.02)PGKEGYIMGNDYGENER.K K.C(+57.02)PGKEGYIMGNDYGENERK.F	Metalloserrulase 18 OS = *Tityus serrulatus* OX = 6887 PE = 2 S V = 1
A0A0C9RP91	89.83	8	3	3	29546	492.9219 418.2051 451.2379	3 2 2	R.LGTVDRQSGPQYR.F R.QSGPQYR.F G.DSGGPLVTR.N	Putative metalloproteinase, TSA: *Tityus bahiensis* Tbah01461 mRNA sequence (Fragmen t) OS = Tityus bahiensis OX = 50343 PE = 2 SV = 1
A0A1S5QN67	87.09	14	4	1	41560	719.3635	3	R.TMTQKKPSGVANAAGLAYYGK.V	Metalloserrulase 20 (Fragment) OS = *Tityus serrulatus* OX = 6 887 PE = 2 SV = 1
A0A0C9RPA3	86.26	10	4	1	43099	487.1898	2	K.VC(+57.02)DEC(+57.02)YK.V	Putative metalloproteinase, TSA: *Tityus bahiensis* Tbah01003 mRNA sequence (Fragmen t) OS = Tityus bahiensis OX = 50343 PE = 2 SV = 1
A0A1E1WVW9	63.40	5	5	2	44401	592.7829 452.7415	2 2	K.FSTC(+57.02)SVENIK.Y K.SDPPFITK.S	Putative metalloproteinase OS = *Tityus obscurus* OX = 122124 0 PE = 4 SV = 1
A0A1Y3BFR2	60.55	4	2	2	36493	433.2403 440.7520	3 2	E.NFRPVQPLNGR.Q R.PVQPLNGR.Q	Carbonic anhydrase 2-like protein OS = *Euroglyphus maynei* OX = 6958 GN = BLA29_002815 PE = 3 SV = 1

a Peptide spectral matching search performed against the Uniprot Arachnida database, using Peaks X software

^b^*m/z* and *z* values correspond to listed ion peptide sequences.

### Evolutionary distance between *T*. *trivitattus* populations from Paraguay and Argentina

**Fig 6:**
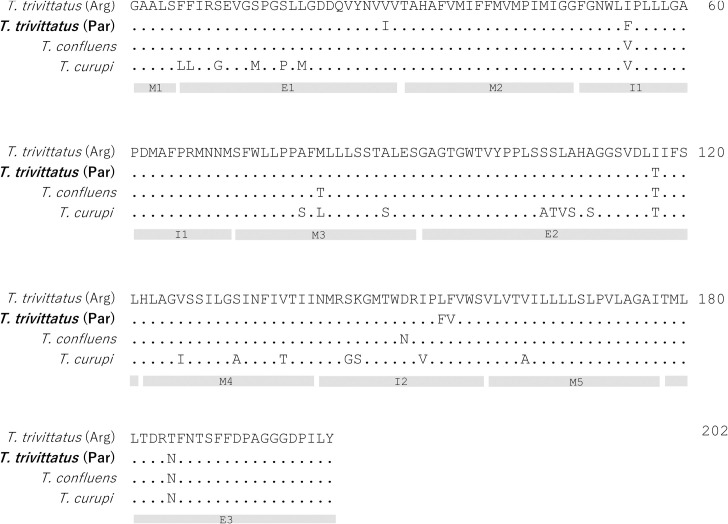
Comparison of Cytochrome oxidase Subunit I N-terminal sequence from *T*. *trivitattus* (Paraguay) population with geographically related *Tityus* spp. Dots indicate identical residues. Bars shaded in gray indicate COI extracellular (E), transmembrane (M), and intracellular (I) regions based on Lunt *et al*. [39].

**Fig 7:**
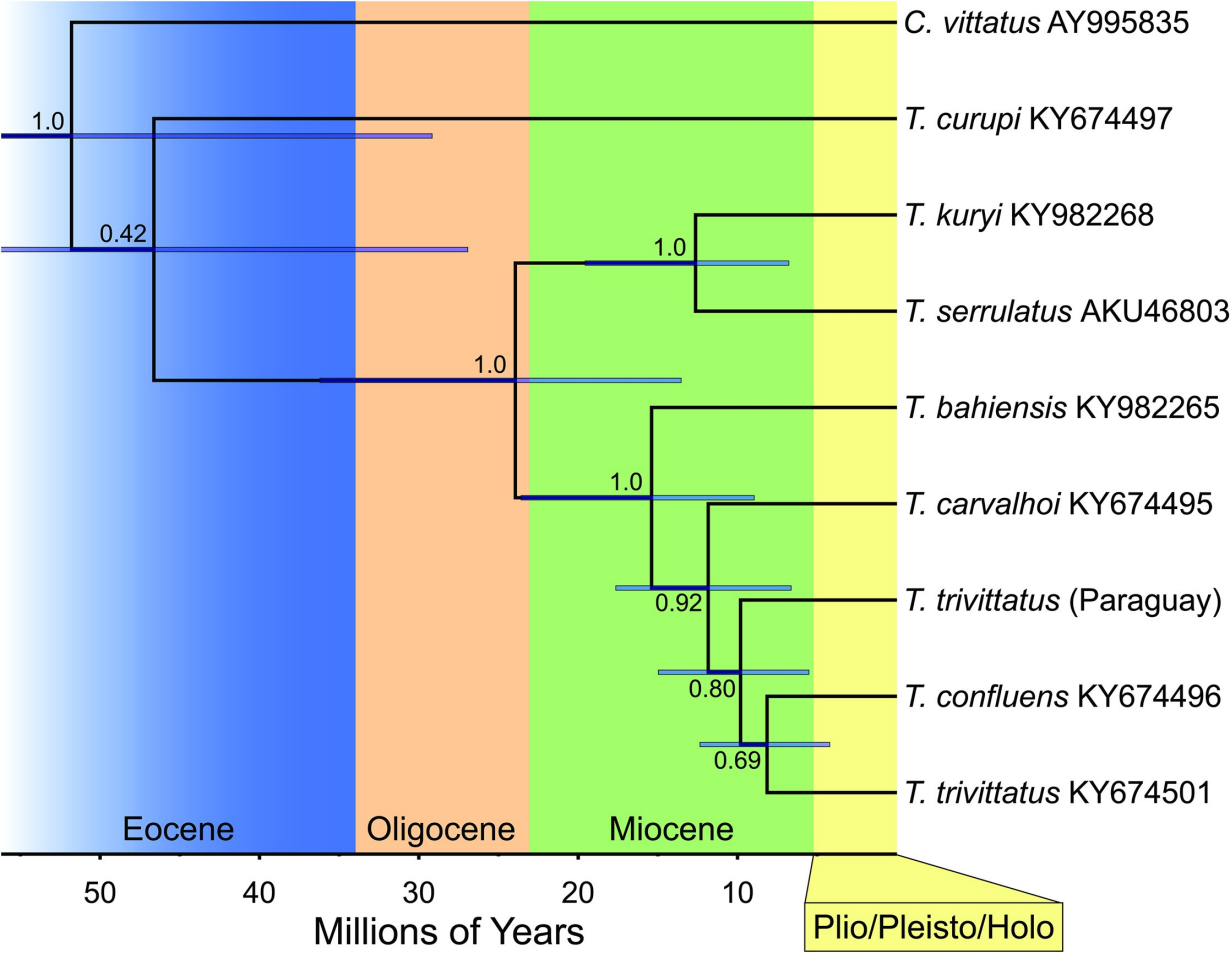
Consensus tree depicting the results of a Bayesian analysis of COI sequence data generated using BEAST for selected *Tityus* spp. from southern South America. Values at nodes indicate posterior probabilities; bars indicate highest posterior density (HPD) values around mean date estimates. Accession numbers are indicated for COI sequences retrieved from GenBank. *Centruroides vittatus* (Buthidae) was included as outgroup.

Considering the variations in venom composition and antigenicity between venoms from conspecific populations of *T*. *trivittatus*, we asked whether significant genetic differences occurred among populations as well. Mitochondrial DNA COI data, which is used for DNA barcoding [38], revealed 6 amino acid replacements between Argentinean and Paraguayan *T*. *trivittatus* populations, whereas the closely related *T*. *confluens* exhibited 5 replacements in the same region (Fig 6). The replacements all occur in highly polymorphic COI areas, particularly transmembrane (M), extracellular (E), and intracellular (I) segments M3, E2 and I2 [39]. In the time-calibrated phylogeny generated with BEAST [40] (Fig 7), the Paraguayan *T*. *trivittatus* is 8.14% divergent (at the nucleotide level) from *T*. *trivittatus* from Argentina, and 8.97% from *T*. *confluens*. *T*. *trivittatus* from Argentina and *T*. *confluens* are 8.47% divergent. Divergence time estimates (using calibration data from [41]) indicate that the *T*. *trivittatus* populations from northern Argentina and Paraguay diverged between the middle Miocene and early Pliocene (~15–5 Million years ago (Ma)).

## Discussion

This study is the first to compare venoms among core (Argentinean) and peripheral (Paraguayan) populations of the noxious scorpion *Tityus trivittatus*. Our results indicate that the population inhabiting urban areas of eastern Paraguay is of potential medical importance, as its LD_50_ value is within the range of other congeneric species associated with lethal envenomations. Table 5 shows a comparison of the calculated LD_50_ with venom lethality values obtained for other *Tityus* spp. in South America in mouse bioassays using the same injection route.

**Table 5:** LD_50_ comparison of venoms from *Tityus* species in assays using 20–22 g mice and intraperitoneal injection route, including 95% CI (in brackets).

Species/Geographic origin	LD_50_ (mg/kg), i.p.	Reference
*Tityus asthenes* (Colombia, Antioquia)	6.08 (5.19–6.98)	[42]
*Tityus pachyurus* (Colombia, Tolima)	4.80 (4.40–5.20)	[43]
*Tityus fuhrmanni* (Colombia,Antioquia)	3.90 (3.00–4.90)	[44]
*Tityus trivittatus* (Argentina, Córdoba)	1.45 (1.15–1.80)	[12]
*Tityus serrulatus* (Brasil, Minas Gerais)	1.30 (0.99–1.65)	[45]
*Tityus trivittatus* (Paraguay, Asunción)	1.19 (0.89–1.71)	This work
*Tityus trivittatus* (Argentina, Entre Ríos)	1.03 (1.00–1.05)	[12]
*Tityus trivittatus* (Argentina, Catamarca/La Rioja)	0.90 (0.60–1.15)	[12]
*Tityus confluens* (Argentina, Jujuy/Catamarca)	0.70 (0.45–1.05)	[46]
*Tityus trivittatus* (Argentina, Entre Ríos/Santa Fé)	0.70 (0.50–1.00)	[12]

**Fig 8:**
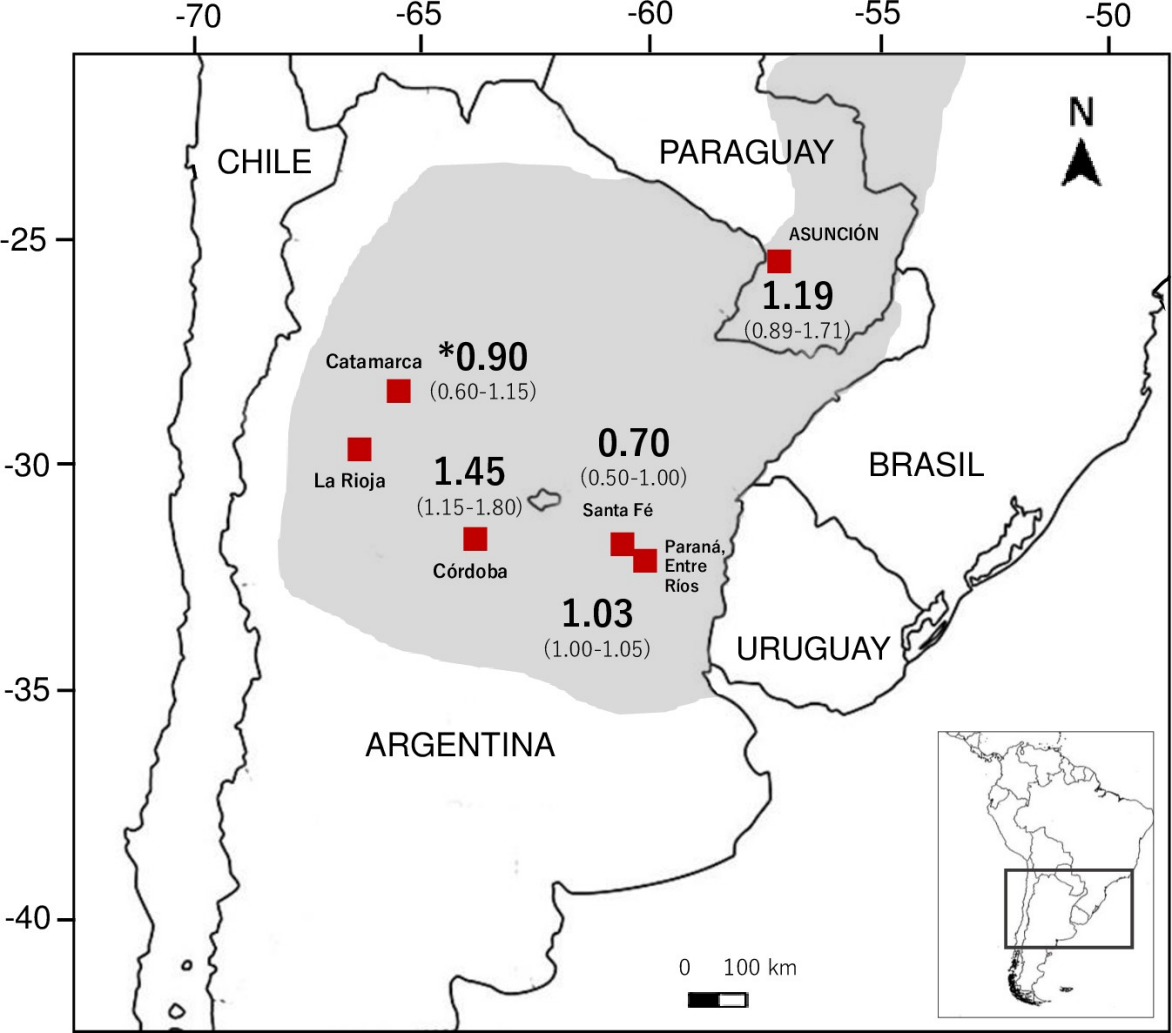
Geographic distribution of *T*. *trivittatus* in southeast South America (based on [11], in gray). Localities (red squares) are shown in Argentina where *T*. *trivittatus* venom lethal medium doses have been determined in mice using the intraperitoneal route of injection (mg/kg, in boldface) [12], including that reported in this study from Asunción, Paraguay (95% confidence limits in brackets). (*, dose reported is from a venom mixture from Catamarca and La Rioja).

Particularly, venom lethal potency for the Paraguayan population is comparable to those reported from the Argentinean provinces of Entre Ríos, Santa Fé, Córdoba, La Rioja, and Catamarca, where *T*. *trivittatus* is prevalent and associated with stings, mainly in children, with incidence rates ranging from 5.62 to 32.51 cases per 100.000 inhabitants [47] (Fig 8). Although the overall risk of mortality per envenomation is relatively low, the wide distribution and the synanthropic behavior of *T*. *trivittatus* make it a significant public health risk [4]. Specimens representing the local Paraguayan population of *T*. *trivitattus* were mainly found in crevices and pipelines inside human dwellings. A population of *T*. *confluens*, a species of medical importance in Argentina [46], also inhabits Great Asunción, but its local sanitary importance remains to be determined.

As severe scorpion envenomations have been reported from Asunción and neighbouring areas in Paraguay [10], it was important to evaluate the neutralizing capacity of therapeutic scorpion AVs from Brazil and Argentina against local populations of *T*. *trivittatus*. These antivenoms are proven therapeutic tools in both countries against human envenomation by *T*. *serrulatus* and *T*. *trivittatus* [9,4]. We did not assay the AV produced in Mexico against species in the genus *Centruroides* as its lower immunoreactivity towards *Tityus* spp. venom components has been demosntrated [1,12]. The antivenom produced against *T*. *discrepans* in Venezuela was not used either considering its low recognition towards venoms from southern South American *Tityus* spp. and that it does not abolish the action of *T*. *serrulatus* NaTxs and KTxs on ion channels [48,1]. Our preliminary assessment using a single dose of antivenom suggested that the Brazilian (anti-*T*. *serrulatus*) AV offered the best protection in the mouse neutralization assay. Eventhough the amount of AV protein used per mouse was similar in these assays in the case of the Brazilian (281 μg) and Argentinean (331 μg) AVs, protection provided by the INPB AV from Argentina was 50% (Table 1). To investigate whether there were variations in AV reactivity that could account for such *in vivo* differences, we carried out immublotting assays. Western blots indicated that a greater number of protein components from both *T*. *trivittatus* populations cross-reacted with components from *T*. *serrulatus* AV, compared to the reactivity observed after probing membranes with the INPB AV, particularly in the region where low molecular mass neurotoxins migrate (< 10 kDa). This could contribute, at least in part, to the higher *in vivo* neutralization provided by the Brazilian AV. Importantly, INPB antibodies recognized components from the Paraguayan population at a lesser extent compared to Argentinean samples, including neurotoxic peptides (< 10 kDa) associated with lethality, which was unanticipated considering that these populations have been historically regarded as conspecific [25,11]. To evaluate quantitatively the antigenic differences between venoms from both populations, titration and competitive ELISA assays were carried out using the INPB AV, which is the only antivenom used in Paraguay to treat envenomed victims [10]. Titration ELISA tests showed that *T*. *trivittatus* venoms from Paraguay and Argentina were similarly recognized by the anti-*T*. *trivittatus* (INPB) AV, probably as a result of the contribution of high molecular mass components (Fig 3A). Tested using the same concentrations in competitive ELISA assays, venom from Paraguay did not reproduce the inhibition curve obtained with venom from Argentina in the ability to prevent binding of INPB antibodies to inmobilized control venom, with 77.2 ± 4.2% antibodies remaining free in solution (Fig 3B). Taken together with the immunoblotting results and *in vivo* data, such partial competition reinforces the suggestion that venom components with significant antigenic differences exist across the geographic distribution of *T*. *trivittatus*, some of which could account for the venom toxicity of the Paraguayan population. In addition, SDS PAGE showed differences in both type and relative abundance of venom proteins between Argentinean and Paraguayan *T*. *trivitattus* (Figs 2 and 4). Previous work has shown that the INPB AV effectively neutralizes venom from Argentinean *T*. *trivittatus* whereas a fourfold amount of Brazilian anti-*T*. *serrulatus* AV is needed for neutralization of the same venom challenge dose [49]. Taken together with our results, these previous findings suggest that southern South American *Tityus* spp. produce toxins antigenically more diverse than envisaged in previous studies [7].

Venom proteins are shared between these populations in the high molecular mass range (20–60 kDa), as determined by SDS PAGE (Fig 4), cross-recognition in blots (Fig 2), and ELISA titrations (Fig 3A). Some of these proteins have been identified in scorpion venoms as metallopeptidases and hyaluronidases, which contribute to the severity of the envenomation process. Some *T*. *serrulatus* metalloproteases are capable of hydrolyzing neuropeptides *in vitro*, releasing mediators that could interact with ion channels and promote indirect neurotoxicity [50]. In particular, a group of scorpion metallopeptidases named Antareases have been postulated to play a role in the development of scorpion venom-induced pancreatitis as they cleave SNARE (N-ethylmaleimide-Sensitive factor Attachment protein Receptors) isoforms associated with zymogen granule membranes in exocrine pancreas, disrupting the normal vesicular traffic [51,52]. Scorpion venom hyaluronidase activity significantly enhances bioavailability of low molecular mass neurotoxins as has been shown in *T*. *serrulatus* [53]. Notably, transcriptomic studies have indicated that proteases are the most abundant transcripts in Brazilian scorpions from the genus *Tityus*, representing 48%, 38%, and 33% of the venom glands transcripts of *T*. *obscurus* [54], *T*. *bahiensis* [35], and *T*. *serrulatus* [54], respectively. Thus, we explored proteolytic (gelatinolytic) and hyaluronidase activities in *T*. *trivitattus* venoms. Fig 4 (right panel) shows a major band with hyaluronic acid-degrading activity migrating between 40–50 kDa, which is in the range reported for other scorpion venom hyaluronidases [53,22]. Distinct hyaluronidases have been reported from *T*. *serrulatus* and *T*. *bahiensis* but little is known about the potential existence of catalytic differences among isoforms [55,35]. Fig 4 (middle panel) shows that the majority of proteins with gelatin-degrading activity were population-specific, with a major component in the Argentinean population migrating around 37 kDa, and a protein around 110 kDa in the population from Paraguay. Differences in the proteolytic enzymes of both *T*. *trivittatus* populations are apparent from these results.

To further explore the differences in protease content between *T*. *trivittatus* populations we subjected the 30 kDa component, which is present at different abundances in these two samples and is within the mass range of other scorpion proteases, to trypsin digestion and proteomic analysis through nESIMS/MS. Sequences of most tryptic peptides derived from both populations matched Brazilian *T*. *bahiensis* and *T*. *serrulatus* metalloproteinases. However, component from the *T*. *trivittatus* Paraguayan population contained peptides similar to additional *T*. *serrulatus* metalloproteinases (metalloserrulases 1, 16, 18, 20) compared with the Argentinean population (metalloserrulases 18, 20). Distinct metalloserrulases have a preference for cleaving neuropeptides with high specificity, implying that they are neuropeptidases with different biological targets and roles in the envenoming process [37]. Considering the differences in venom gelatin zymograms and electrophoretic mobility, it is feasible that proteolytic proteins with differential properties exist in these *T*. *trivittatus* populations that could influence their toxicity. However, a full proteomic/transcriptomic study is needed in both cases for a proper comparison of their proteolytic components.

To gain further knowledge into the protein composition of both *T*. *trivittatus* populations, MALDI TOF MS was used to determine their venom fingerprint in the NaTx and KTx ranges (Fig 5). In the NaTx range, the populations shared components of masses 6606.1 Da and 6940.2 Da. The latter closely resembles the calculated mass for toxin Tt1g (6938.12), a β-toxin isolated and characterized from the Argentinean *T*. *trivittatus*, which acts on the sodium current activation component in excitable tissues [7]. The fact that three components in NaTx mass range were unique to the Paraguayan population and six were exclusive to Argentinean *T*. *trivittatus*, together with the observation that no shared components were detected in the mass range of antimicrobial peptides or KTxs provides additional evidence for their toxinological divergence. Identification of population-specific *T*. *trivittatus* NaTxs by proteomic analysis is currently ongoing. Considering that NaTxs are the most lethal components of buthid scorpion venoms [8], such identification is crucial for the design of therapeutic antivenoms that aid in the neutralization of specific toxic components of Paraguayan *T*. *trivittatus*. Previous studies have shown that antibodies prepared against recombinant NaTxs effectively neutralize venom lethality and that NaTxs could be used as immunogens in antivenom manufacture [56].

In regard to their toxinological differences, we asked whether there could be evolutionary differences between these *T*. *trivitattus* populations as well. Amplification of a fragment encoding COI, which is used for DNA barcoding, allowed sequence analysis, both at the amino acid and nucleotide levels. A Bayesian analysis revealed that the Paraguayan and Argentinean *T*. *trivittatus* populations are 8.14% divergent at the nucleotide level (Fig 7) and may represent distinct species as this is within the range of COI divergence for other scorpion species in the family Buthidae [57,32]. Additionally, *T*. *trivitattus* from Paraguay exhibits more amino acid replacements in this COI segment with respect to the Argentinean population in comparison to *T*. *confluens* (Fig 6). Divergence time estimates for these populations correspond to the middle to late Miocene, between 5 and 15 Ma, based on our time-calibrated phylogeny. This timeframe overlaps closely with the estimated age of the inland sea that existed in southern South America, named the Paranaense sea, between 15 and 13 Ma [58]. The sea occupied most areas of northern Argentina and Uruguay [59], and could have isolated the genetically divergent Paraguayan population of *T*. *trivitattus*. The same mechanism has been postulated for frogs in the genus *Lepidobatrachus*, armadillos in the genus *Calyptophractus*, and geckos in the genus *Homonota* [60–62]. Given the divergence date estimates, genetically differentiated *Tityus* populations could have originated by vicariance as Miocene marine incursions along the Paraná river basin fragmented their ancestral range.

*T*. *trivittatus* was described in 1898 based on specimens collected in San Salvador (presently in Guairá department, eastern Paraguay), 120 km southeast from Asunción, within the current distribution range for this species in Paraguay [63,25]. As such, our sampled Paraguayan population represents *T*. *trivittatus sensu stricto* which warrants further research to uncover the true taxonomic identity of the supposedly conspecific population inhabiting northern-central and eastern Argentina, historically identified as *T*. *trivittatus* [11]. Morphological differences between these populations would confirm our findings.

Taken together, our results suggest that further venom and taxonomic diversity exists in southern South American *Tityus* than previously thought. Further research is being carried out in our laboratories to determine the true extent of the toxinological relationships between *T*. *trivittatus* populations inhabiting urban areas in Paraguay and its synanthropic Argentinean and Brazilian congeners, both in venom composition and function. Importantly, such studies would aid in the design of more effective therapeutic tools against scorpionism in the region.

## Supporting information

S1 Data Venom reactivity of *Tityus trivittatus* populations from Paraguay and Argentina towards INPB (anti-*T. trivittatus*, Argentina) horse antibodies measured at 495 nm using ELISA titrations. (XLSX)

S2 Data Binding of free antivenom (INPB, Argentina) (%) to immobilized *Tityus trivittatus* (Argentina) venom in the presence of competing *T. trivittatus* venoms from Paraguay and Argentina. (XLSX)
